# Satellite clinics in academic ophthalmology programs: an exploratory study of successes and challenges

**DOI:** 10.1186/1471-2415-13-79

**Published:** 2013-12-14

**Authors:** Irene C Kuo

**Affiliations:** 1 Cornea and Refractive Surgery Services, Wilmer Eye Institute and Wilmer Eye Institute at White Marsh, Department of Ophthalmology, Johns Hopkins University School of Medicine, 4924 Campbell Blvd. #100, Baltimore, MD 21236, USA

## Abstract

**Background:**

Major academic ophthalmology departments have been expanding by opening multi-office locations (“satellites”). This paper offers a first glimpse into satellites of academic ophthalmology departments.

**Methods:**

Leaders of seven medium to large, geographically diverse departments agreed to participate. One- to two-hour phone interviews were conducted to assess the features of their satellite practices.

**Results:**

Success as clinical entities, profitability, and access to patients were stated goals for most satellites. In approximate descending order, refractive surgery, retina, oculoplastics, and pediatric ophthalmology were the most common subspecialties offered. Faculty staffing ranged from recruitment specifically for satellites to rotation of existing faculty. Except for a department with only one academic track, satellite doctors were a mix of tenure and mostly non-tenure track faculty. According to these department leaders, scholarly productivity of satellite faculty was similar to that of colleagues at the main campus, though research was more community-based and clinical in nature. Fellowship but little resident education occurred at satellites. Though it was agreed that satellite practices were integral to department finances, they accounted for a smaller percentage of revenues than of total departmental visits.

**Conclusions:**

Satellite offices have offered access to a better payor mix and have boosted the finances of academic ophthalmology departments. Challenges include maintaining collegiality with referring community physicians, integrating faculty despite geographic distance, preserving the department’s academic “brand name,” and ensuring consistent standards and operating procedures. Satellite clinics will likely help departments meet some of the challenges of health care reform.

## Background

In recent years, major academic ophthalmology departments have been expanding by opening multi-office locations (“satellites”). To our knowledge, there is little peer-review literature on satellite offices in academic medical departments in general, [1,2] let alone in ophthalmology. This paper offers a first glimpse into these practices.

Ophthalmology departments were in expansion mode in the 1970s and 1980s. Under the auspices of a very young National Eye Institute, the first comprehensive assessment of major needs and opportunities in vision research in the United States was published [3]. Many eye institutes and laboratories also were constructed [4]. However, major changes occurred in delivery of ophthalmic care in the late 1980s [5-7]. In 1987, Medicare granted surgery centers an 18.7% rate increase while drastically curtailing hospital outpatient surgery payments [8]. One result was that academic ophthalmology departments began to expand beyond their typical urban settings.

On the centenary of Abraham Flexner’s report*, Medical Education in the United States and Canada*[9], an article described the shift in focus of faculty practice plans in the 1980s to the operation of satellite centers and ambulatory care facilities and how “physicians were recruited into the medical school whose main responsibility was clinical care and who were not intimately linked to its education and research missions” [10]. Bentley et al. provided a non-department specific description of the modern academic medical center: “Satellite centers, which offer a variety of primary and specialty care serves, are being established to increase patient referrals to the main campus, as service areas are expanded beyond historical boundaries” [2]. Do these findings apply to ophthalmology departments? The purpose of this survey was to offer an exploratory, descriptive first glimpse into satellite practices of academic ophthalmology departments by ascertaining the views of department leaders--to discern common themes of successful satellite practices at various programs and common programmatic issues that need to be addressed.

## Methods

As this project was a pilot study of satellite clinics of major academic ophthalmology programs, interviews of department leaders were deemed a first step to uncover common themes. Johns Hopkins Medicine Institutional Review Board X determined that this research qualified for an exemption under Title 45 Public Welfare Department of Health and Human Services Code of Federal Regulations Protection of Human Subjects 46.101(b). Academic ophthalmology departments from large metropolitan areas (population range: 1.7 million to 9.8 million, 2010 United States census) on the East Coast, West Coast and the Midwest were selected based on purposive sampling, [11] geographic diversity, information that they had satellite offices, and a strong impression that their chairs had the potential to provide relevant, diverse data pertinent to the research question. Seven departments were selected, and their chairs, understanding the results might be published, agreed to participate when contacted. The author (a subspecialty-trained ophthalmologist hired by one department to open a satellite clinic and served as its medical director) conducted phone interviews with chairs, except when they recommended a vice-chair or chief executive officer; questions were not provided in advance, anonymity was promised, and interviews (which were not recorded) lasted 1–2 hours long. The questionnaire consisted of approximately 20 questions (see “List of questions asked of department representatives”); it was not validated, given the nature of this pilot study. Quotations that were detailed, reflecting either common sentiment or contrasting opinions, were included. When answers were ambiguous or not provided, a follow-up phone call was made.

### List of survey questions asked of department representatives

How many satellites does your department have?

Why do you have satellites? What is/was the strategy behind developing them?

What was the reaction of community ophthalmologists when you built these satellites? How did you respond to their concerns?

Where are the satellites located? How far is the farthest one?

Do your satellites offer multispecialty care?

How is the academic mission fulfilled? In other words, are teaching, research, and patient care conducted at all satellites? If not, what combination?

What kinds of doctors are at your satellites—junior, senior, or mix?

Are there different academic tracks in your institution? If so, in which track do most satellite doctors fall?

Who decides which doctors go to satellite clinics?

Is there a director for each satellite?

In which academic track do most satellite directors fall?

For what percentage of total visits to your institution is accounted by your satellites?

For what percentage of total revenue is accounted by your satellites?

How do you evaluate the success of a satellite?

Have you had to close any satellites?

What are the successes of having satellites?

What are the challenges?

## Results

One department of the seven was excluded because it had only Veteran’s Administration and county hospitals, which, being hospital-based, by definition are not satellite practices. No two departments were in the same metropolitan area. Reasons for opening satellite clinics included inability to expand at the main campus; the perception that the main campus’s (urban) location was a disadvantage; proximity to philanthropic sources (which raised funds to build two satellites in one department); ability to meet suburban population growth; and agility to meet the challenges of managed care. All leaders confirmed a different payor mix at satellite clinics vs. the main campus. Said one representative, “Fear is a great motivator. Practice maintenance was the most important reason we opened satellites. It was reasons 1 through 10. We opened satellites because we did not want to be in [financial] 2^nd^ place”.

### Location and number of satellites

Locations ranged from within 5 minutes of the main campus to about 100 miles away for satellites of three departments. A leader who opened his department’s first satellite in the early 1990s, recalled, “We asked if the satellite would fulfill the missions of an academic institution—patient care, research, teaching. There was an element of opportunism. We asked was [our medical school] parent there with other departments? This provided access to patients. Were personnel there? [Aside from money], we needed space, referrers, and staff. When we got a marriage of two of three factors, it became a growing concern, an ‘entity’”.

The number of satellites (range: 1–6) was not correlated with the total number of departmental clinical encounters or with the size of the surrounding metropolitan area. In fact, in this survey the departments with the fewest and most satellites were located in cities of similar population. Another department had twice as many satellites as a similar-sized department in a different city.

### Subspecialties and faculty

Satellites of all 6 departments offered multiple subspecialties, but not the same ones at every satellite. Satellites of two academic centers did not offer comprehensive ophthalmology to preserve the referral base of comprehensive ophthalmologists; some of their satellites offered only retina. The most common subspecialties at satellites were (in descending order) refractive surgery and retina (close approximation), oculoplastics and pediatric ophthalmology, and cornea and glaucoma.

Most satellite clinics were staffed by junior faculty with a few senior level faculty. However, one leader explained that satellites were staffed by senior level faculty in order to “ease the strain with the community.” In another department, the profile has changed; all faculty at its first satellite were senior level, but over time satellites have become staffed by junior faculty. Two chairpersons were seeing patients at satellites.

One department had a single academic track; the rest had multiple tracks, the main ones being “traditional”/”tenure” track (the “triple-threat” academician with grant support, teaching and patient care responsibilities) and the “clinical care”/”clinician educator” track. Regarding recruitment, “most faculty [at satellites and at the main hospital] are hired as clinician educators unless they are fully bred as a clinician scientist with lab interest,” explained one representative.

In a department with close to 10 satellites, tenure-track faculty had a high representation at satellites closer to the main campus, whereas clinician-educators populated outlying satellites. Except for the department with one satellite, each satellite had a medical director, usually a clinician educator. One leader stipulated when opening his first satellite 20 years ago, the medical director “had to maintain patient volume and command respect from referring doctors. Our doctor could not be inferior to referring doctors and had to be better than the local practitioner”.

Faculty staffing could involve recruiting specifically for a satellite, assigning current faculty or having them volunteer to go. One representative stated, “There is a better payor mix at satellites, so you want faculty who will work hard and be willing to compete against good people in the community. We don’t want doctors to view joining a satellite as a way to get into [our department]”. Another department leader declared, “We don’t hire specifically for a site. We are part of a team. We accommodate the needs of the whole institution. Faculty have a vested interest in the well-being of the eye institute and buy into the idea of its success. We want to create ownership”.

### Education

Trainee education occurred at some or all satellites, but fellows were present more so than residents. There was no “resident clinic” at any satellite anywhere. Only one chair stressed his expectation of teaching: “Faculty who are full-time at satellites still have to go to the main hospital to teach”. Another leader admitted that teaching at a satellite could impede its efficiency and presumably its attractiveness to patients. Summarized one chair: “Eventually a satellite has to fulfill the academic mission, but in order for the satellite to survive, it has to be noted for providing good patient care”.

### Finances

As a share of total revenue generated by the respective department of ophthalmology, satellites accounted for a range between 10% (program with only one satellite) to 50% (program with the most satellites) (Table 1). Numbers were provided by department leaders or published in ophthalmology newspapers. As a share of total visits, satellite visits ranged from less than 5% (program with one satellite) to 80% (program with the most satellites). Except for the program with one satellite, satellites accounted for a smaller percentage of total revenue than of total visits. The ratio of percentage of total revenue to the percentage of total visits ranged from 33% (program with a very large number of satellites) to 200% (program with one satellite). At the program with the most satellites, this ratio was 63%. One reason given for the disparity between visits and revenue was that cases at the main campus tended to be more complex and tertiary, leading to more surgical revenue. Information from one department was not forthcoming.

**Table 1:** Comparison of academic programs in terms of volume of clinical encounters and operating room procedures, number of satellites, and contribution of satellites to total revenue and total number of visits in fiscal year 2010

**Academic program**	**Approx number of annual clinical encounters**	**Approx number of operating room procedures**	**Number of satellites**	**% of total revenue accounted for by satellites (G)**	**% of total visits accounted for by satellites (H)**	**Ratio of % of total revenue to % of total visits (G/H)**
A	60,000	2,500	1	>10	<5	>2
B	130,000	8,000	9	<33	>33	<1
C	179,000	7,500	9	25	50-55	<0.5
D	220,000	12,300	4	12	20	0.6
E	300,000	50,000	12	50	80	0.6
F	N/P	N/P	5	30-40	N/P	N/P

N/P = not provided.

### Successes and challenges

Department leaders credited satellite clinics for providing access to more patients: “There are lots of patients, a lot of revenue stream”. One representative said, “The successes of satellite clinics lie in the areas we know we can be financially stable,” like community-based research. Said one leader, “As the department expands, it seems that fewer doctors want to stay at the [main campus]. They want to go where the patients are. Also the staff tend to be more efficient and independent at satellites”. One representative claimed that patients benefited by not having to travel to the inner city for consultation. Though most departments did not track nor could they estimate the numbers, leaders sensed that referrals to the main medical campus had increased with ophthalmology satellite presence; one representative claimed a 10% increase in referrals to the ophthalmology department at the main campus as a result of satellite presence the past 10–15 years.

The success of satellite clinics, however, comes with challenges. Given that satellites can be up to 100 miles away from the main campus, several leaders voiced concern about integrating faculty members and having consistent standards and operating procedures throughout the department. Satellite doctors may not communicate face-to-face with department colleagues often; mentorship may suffer. Also, research labs and core resources were usually located at the main campus as were residents; fulfilling the education and academic missions might prove challenging as the number of satellites continued to grow. “We should find out how (other departments) keep satellite doctors involved,” said one representative. A related issue (but mentioned by only one representative) was how to preserve the “[academic] brand” and not dilute it when opening remote satellite clinics.

Competition with community (referring) ophthalmologists posed another challenge. Only one leader said there was not much antagonism—at least none of which he was aware. “The scope is so limited; we are not offering all specialties”. However, most representatives were aware of antagonism from community ophthalmologists at the start, evolving into wariness as satellites grew in number. “We discovered that local eye doctors voted 100 to 1 against our presence,” recalled a representative whose first satellite opened 20 years ago; all faculty were required to go. “At the time, there were no comprehensive ophthalmologists on faculty. Our goal, therefore, was to be a consultation service to the community, not to be in the hospital and not to do surgery,” he continued. In response to community antagonism, another department communicated that it was placing only specialists in its satellites. “However, if a patient comes to us with a cataract, we are not going to avoid doing the surgery,” the department leader said. Two departments gave community ophthalmologists the option to buy into satellite practices or use a satellite surgery center. Another leader explained that the department told community doctors that satellites were part of the dean’s initiative, not the doing of the department itself.

Five departments were building more satellites at time of writing—all outside the geographical center, with one being built 50 miles away from the main campus. The representative of the sixth program (the smallest department and possessing only one satellite) was unsure about expansion: “We have to think carefully. There is potential risk with any growth, especially in this uncertain time with health care reform. Every satellite represents a financial risk as it entails financial investment. When you open a satellite, you look for confirmation that what you did was good”. This leader, however, admitted, “I believe that we would have enjoyed greater success had we opened the satellite sooner”.

Similarities and differences amongst the programs are summarized in Table 2.

**Table 2:** Summary of similarities and differences amongst six ophthalmology departments with satellite offices (number of departments)

**Similarities amongst majority of programs**	**Differences amongst programs**
Suburban location of satellites (6)	Number of satellites relative to size of the department (as measured by clinical encounters)
“Patients do not want to travel as far as they used to in order to see their doctor at the medical center.”	
Lease rather than purchase space for satellite offices (6)	Satellites developed de novo vs. acquired (e.g., department buys community practices)
Satellites led by clinician or clinician-educator (5):	Type of doctors at satellites:
“Time carved out for administration detracts from research and clinic”	- hiring specifically for satellites (doctors with “private practitioner” mentality) vs.
“We need people who can build a practice, clinicians who can provide good consultations”	- rotating existing faculty members vs.
“They have to be responsive to referring doctors’ needs”	- hiring by a subspecialty division then rotating faculty to satellites
Satellites staffed predominantly by junior faculty (5)	Senior doctors at satellites closer to medical center
	Academic rank of faculty members at satellites
Type of specialties offered in approximate descending order (6): refractive surgery, retina, oculoplastics, pediatric ophthalmology, cornea, glaucoma	Decision to offer comprehensive ophthalmology at satellites; to have optometrists at satellites
Revenue/visit is less at satellites than for over all department (5)	Some departments have “hub and spoke” model (surgical and/or more difficult cases are shunted from satellite to main medical center)
Better payor mix at satellites (6)	
Concern about integrating faculty members, maintaining cohesive group of faculty (4)	Concern about mentorship
Perceived strain with community ophthalmologists (4)	Providing consultation to community doctors vs. competing directly with them (by offering “general ophthalmology” at satellites, for example)
Lower staff/patient ratio at satellites compared to main medical center (4)	
Teaching of fellows, not residents, at satellites; no resident clinic at satellites (5)	Types of research/scholarly pursuits
	-success in “clinical research and community-based research projects.”
	-“Research coordinators can conduct clinical trials. We want to make [satellite doctors and staff] part of the overall academic mission.”
	-“Every faculty member has to be plugged into teaching.” Even full-time satellite faculty have to teach at the main hospital
Financial potential or constraints are most important determinants in opening or closing a satellite; financial benchmarks (6):	Concern about preserving academic “brand” as open more satellites
	Patient satisfaction, physician/staff performance, infection control, tracking surgical complications
“A satellite is a total business decision”	
Increase in number of visits to eye department at main hospital as a result of satellites (3)	

## Discussion

To our knowledge there is no literature on satellite offices in academic ophthalmology. Satellite offices have offered access to a better pay or mix and have boosted the finances of academic ophthalmology departments. In fact, the decision to build or continue a satellite practice appears to be nearly entirely financial, distinguishing it from a department’s other divisions. The fact that satellites do not offer all sub-specialties may lower overhead and lead to more referrals to the main campus. Areas to be addressed include trainee education at satellites; integration and mentorship of satellite faculty; relationship with community physicians; [1] and preservation of the department’s academic “brand”.

Understandably, the triad of patient care, teaching, and research forms the mission of all academic medical institutions. Direct patient care, however, is not conducted in basic science laboratories. Moreover, limited research is conducted in most resident clinics, which, being centerpieces of education, usually are not held to the same financial standards as other divisions. Therefore, it may not be valid to hold satellites to the standards of the over all department as one criticism has been that they do not contribute to the education mission.

The financial contribution of satellites most likely supports this mission as well as others of the department and medical school. More than 100 years ago, however, Flexner opposed this model of full-time faculty for fear that medical education would suffer when faculty were pressured to engage in clinical practice to support the institution and became the “scientifically dead practitioner” [2,9]. This concern was voiced again mid-century by the Association of American Medical Colleges, which described the inherent danger of medical schools applying pressure on faculty to increase clinical revenues “in order to help finance the overall activities of the institution” [12]. One limitation of this paper is that by design, it represents the opinions of several department chairs and vice-chairs. Their views may not be shared by their own faculty or by other leaders. Undoubtedly, faculty input will be important as departments ponder how to integrate satellite clinics in the academic mission.

Issues of faculty integration, academic progress of satellite doctors, and “brand” preservation, (all raised by a minority of department leaders), would be worth further investigation as they pertain to academic identitity. Although representatives asserted that satellite faculty were engaged in research, publications, and teaching, the retention rate of satellite faculty (many of whom are junior level clinician-educators), their rate of promotion, and time to promotion were outside the scope of this study. These rates could be lower if mentorship and interaction with fellow faculty are lacking; compared with other tracks, clinician educators at one major institution were of lower rank and were less satisfied with progress toward academic promotion. [13] Concerns about academic integration and dilution of the department’s academic reputation were not widespread amongst these leaders, but may grow with satellite expansion. Academic centers have evolved from providing tertiary eye care alone to including general eye care, which may affect interactions with referring general ophthalmologists and the research focus of faculty.

Data saturation was achieved for the survey questions. Some differences that were uncovered were number of satellites relative to department size, academic track and rank of satellite doctors, and decision to offer comprehensive ophthalmology. The number of satellites of a given department may reflect local conditions like regional competition and presence of managed care. Revenue per visit at satellites was usually less than for the over all department, perhaps reflecting lower complexity visits and/or lower reimbursement for the same in-hospital procedures. However, some aspects of overhead, like staff/patient ratio, may be lower at satellites.

Growth of satellite practices may help academic departments shoulder financial risk better than can a solo practitioner or a small group practice when participating in the Medicare Shared Savings Program vis a vis Accountable Care Organizations (ACOs). Since specialists will have to work closely with primary care providers to obtain patient referrals and to receive larger shares of cost savings, some believe that ACOs will allow large groups to be better positioned to participate in the savings program [14]. Until the effects of this initiative are known, it remains that large entities are often able to negotiate better rates from carriers than are small practices.

## Conclusions

Satellites appear to deliver on one aspect of the tripartite mission—patient care. Aided by payor mix, high-revenue subspecialties, and increased efficiency, they contribute to department revenues. Examining issues raised by this study may help departments ensure they are meeting their mission in a balanced fashion and help ensure satellite and department success. Because satellites can account for a large proportion of a department’s total patient encounters (and even larger proportion of its *new* patient visits), this pilot study may lead to a larger, more comprehensive and systematic study of ophthalmology clinical practice and organizational behavior. Such study would benefit from a representative sample of staff and faculty members, not just department leaders.

## Competing interest

The author has no competing interest.

## Pre-publication history

The pre-publication history for this paper can be accessed here:

http://www.biomedcentral.com/1471-2415/13/79/prepub
